# Necessity, Entailment, Shared Agonism

**DOI:** 10.1007/s10699-020-09745-2

**Published:** 2021-04-21

**Authors:** Dominic Smith

**Affiliations:** University of Dundee, 3.10 Tower Extension, Nethergate, Dundee, DD1 4HN Scotland, UK

**Keywords:** Necessity, Contingency, Transcendental, Agonism, Postphenomenology, Education

## Abstract

This short paper offers a series of responses to Jochem Zwier and Timothy Barker’s comments on my extended paper ‘Taking Exception: Philosophy of Technology as a Multidimensional Problem Space.’ Part one responds to questions concerning the modality of the renewed understanding of the theme of the transcendental that was argued for in my initial paper: I argue for the deep *contingency* of such a move, against any sense that it is *necessary.* Part two takes this consideration of modality further, considering the *possibilities* that a renewal of the theme of the transcendental stands to offer philosophy of technology today. I argue that the contingency of our contemporary sense of the transcendental can be precisely what makes it valuable. Whereas parts one and two turn on incisive questions posed by Zwier, part three closes by reconsidering the claims for a ‘multidimensional problem space’ offered in my initial paper. In response to an acute insight from Barker, I close by arguing that philosophy of technology’s problem space should be explored in terms of a notion of ‘shared agonism’.

Let me thank Jochem Zwier and Timothy Barker. Their papers demonstrate incisive engagement with mine, and are critical and generous in equal measure.

What struck me was how these papers dovetailed. I had been worrying about what this response paper might look like, but Zwier and Barker’s clarity solved this for me.

Zwier raised two big questions, with interesting sub-questions: ‘[W]hat exactly necessitates the … *rehabilitation* of the transcendental?’ and ‘[W]hat does Smith’s *renewal* of transcendental philosophy imply and entail?’ (Zwier, this issue). Barker raised a closely related issue: does my appeal to the transcendental risk determinism? Additionally, Barker posed an issue that pinpointed something I had been struggling to express in the final part of my paper: agonism. I will attempt to address each of these points, and as many others raised by Zwier and Barker as possible.

## Necessity

Zwier’s first big question is ‘what exactly necessitates the … *rehabilitation* of the transcendental?’ In response, a core presupposition of my paper is this: *nothing* necessitates it.

Some of Zwier’s remarks suggest, in the form of judicious ‘Devil’s Advocate’ points, that engaging the theme of the transcendental may be unnecessary in the sense of ‘redundant’ or ‘superfluous’. This is because well-established approaches in philosophy of technology, such as postphenomenology, may already be doing it. As Zwier puts it in this excellent passage:

[W]ouldn’t a postphenomenological scholar argue that transcendental questions *are* in fact developed [in postphenomenological analyses], but in a deliberately limited way, for instance by focusing on how technologies make a particular experience of the world possible, how technologies make particular scientific objects possible, etc.… My question to Smith would be which transcendental motives are blocked in such [postphenomenological] accounts, and whether such a blockade exists de facto or *de jure* (Zwier, this issue).

I will discuss more approaches than postphenomenology, but let me focus on it first. There can be no doubt that transcendental questions are developed in postphenomenology, on at least two levels: *logically*, it enquires into how particular artefacts act as conditions for the possibility of given experiences and objects; *historically*, its core thinkers (Husserl and early Heidegger) sit very much in the post-Kantian lineage. On both levels, however, I think postphenomenology is rather too ‘deliberately limited’. Logically, it is often quite classically phenomeno-*logical* and anthropo-*logical* in its choice of artefacts and case studies. Historically, the role of the early Heidegger and Husserl within postphenomenology means, I think, that it tends towards a form of ‘small-c’ conservatism, where it is unable to see new emergences in philosophy of technology except through this lens.^1^

To answer Zwier’s question directly: postphenomenology assuredly has a *de jure* sense of the transcendental that it de facto blocks on at least two levels (stated differently: that it represses). There can be no doubt postphenomenology has made a virtue of this: its analyses of conditions of possibility are fine-grained, informative and well-designed, and its typography of relations (embodiment, hermeneutic, alterity, background….) is insightful and helpful in certain (deliberately limited) cases. It’s just that postphenomenology can also be quite staid in its approach, and informed by a rather repetitive set of core thinkers.^2^ To be sure, there are approaches drawing on postphenomenology that go beyond this. For instance: Malafouris’ work with Ihde (2019), Irwin on digital media (2016), Rosenberger on ‘callous objects’ (2017), and Selinger’s work (Frischmann and Selinger 2018). What I take such work to be engaged in—although none of the authors would likely put it this way—is the introduction of methods and concepts that exceed the usual range of postphenomenological desiderata, and that de facto push the scope of a *de jure* sense of the transcendental that postphenomenology assuredly does have.^3^

This leads me to the part of Zwier’s question concerning which transcendental motives postphenomenology blocks. Put simply: it blocks the motive to ‘get metaphilosophical’ or ‘go meta’.^4^ I am now imagining a postphenomenologist who would respond that there are very good reasons to block this: they might object that it’s not ‘deliberately limited’ enough, ‘too postmodern’, that it invites ‘useless speculation’, or that it presupposes extensive knowledge of the history of philosophy when attention to artefacts before us is what is really required. Let me show how this might overlook something important.

I cited this extract from Floridi (2019: 204):

[W]hat interesting systems one may design… is a matter of talent, intuition, hard work, opportunity, good fortune, free thinking, and imagination, and many other variables that are hard to pin down. As Donald Schon correctly put it, the designer (or indeed the logician and the philosopher and anyone who creatively designs solutions) is:

like a chess master who develops a feeling for the constraints and potentials [affordances…] of certain configurations of pieces on the board.

In my paper, I emphasised how this point holds for systems of technological affordances, and sought to show how a focus on exceptional technologies as ‘interesting systems’ might take us beyond Floridi’s use of the chess example. What I also hinted at (footnote 8) but want to emphasise here is that all this also holds for systems of concepts and their relations (that is: philosophies).

I hold that a less ‘deliberately restrained’ tendency to ‘go meta’ in philosophy can productively involve a more developed sense of the transcendental that can usefully zoom in and out on the intra- and inter-relations between diverse philosophical systems and their conditions of possibility.^5^ I also hold that this can be a good motive for philosophy of technology to develop, irrespective of how inclined to reflect on technologies any particular philosopher or philosophical system under consideration may have been (see Smith 2018: 45–53). This is because, in line with the elaboration offered on Floridi’s above quote in my initial paper (but in a different direction), such an exercise can, I think, act as a way of developing the habits of thought that allow us to better conceive, clarify, critique and create technological artefacts and systems of all shapes and sizes.

It might be useful to consider Barker’s point about determinism here. His worry, if I understand it correctly, is that such a tendency to ‘go meta’ might itself be deterministic:

[I]f we locate the transcendental with real but not actual processes, does the transcendental become another name for invisible technical processes? And then, are we rehashing a type of deterministic argument? (Barker, this issue).

The problem with ‘going meta’ in the way outlined above is that it might seem to necessitate an extremely crude form of Hegelianism. For Hegel, famously, ‘the real is the rational’ (2003). Is it the case, then, that I think we should be studying all sorts of philosophical ‘systems’ and their histories to arrive at a final ‘meta system’ that rationalises and determines all the other systems (whether technological or philosophical)?

If a rehabilitation of the transcendental was ‘necessary’, according to a logic the events, objects, and implications of which could be fully thought out in advance, it would inevitably tend towards this kind of (hellish) approach. As I put it at the beginning of this part, however, what I am after is ‘unnecessary’. In saying this, I am not trying to be overly clever with words, and I don’t mean that the approach I am forwarding is redundant or superfluous. I mean that it is *desirable* and *possible* as something fundamentally *historically contingent* and open to ‘*play’* (to use Barker’s excellent term).^6^

In fact, I take Barker’s paper to include an excellent example of the kind of ‘going meta’ involved in a developed sense of the transcendental, but in an unexpected (yet highly ‘actual’) direction:

Let’s take television as an example: The images of the television, the dramas, comedies, news reports, political debates, sporting events, all involve multiple actors, agents, production companies, industry protocols, and so on, each with their own axe to grind. But prior to all of this is a media history of technical developments involving Nipkow disks, flickering lamps, selenium, Baird’s televisor, the work of Marconi, EMI, Bell Labs amongst many others, as well as the development of the electronic binary computer. Although these things are completely invisible in the contemporary television image, they provide the set of conditions for the possibility of representation and hence the possibilities for television discourse (Barker, this issue).

Barker notes in relation to this example that it is his ‘…first instinct to try and ground the transcendental in the real set of technical conditions upon which any mediation is based’ (Ibid.). My first instinct when presented with the example is to be fascinated by it, and to try to understand and learn from it. My second instinct is this: to acknowledge that the simultaneously wide-ranging and fine-grained approach Barker applies to the complex *actual* conditions of television as a technical system might also be applied to the conditions of any given philosophy as a conceptual ‘system’, and that both these approaches might have interesting things to say to one another, and to develop a sense of philosophy of technology as a many-faceted problem space.

When I discuss a sense of the transcendental, it is just this kind of cross pollination and play in considering the conditions of what is contingently and actually ‘given’ I have in mind. I have sought to show this through a consideration of Kant (Smith 2015). Just as Barker’s example might have been radio rather than television, however, nothing logically ‘necessitates’ this. What *suggests* it is Kant’s status as an interesting nexus in the history of philosophy, and what makes it *possible* and *desirable* is the opportunity it affords to show how philosophy of technology might be opened up and made more dynamic as a field.

This allows me to respond to an important point raised by Zwier: ‘does this mean that Kant’s a priori conditions of possibility can and must themselves be traced back to [what, drawing on Floridi, Smith calls] their ‘conditions of feasability’?’ (Zwier, this issue). I really like the emphasis on feasibility here, and a proper response would require a stand-alone paper. The short (and overly compressed) response is this: such an operation *can* happen and would, I think, have very interesting things to tell us; the sense in which it *must* happen, however, ought to be perpetually open to *dispute*. In fact, it is just this ‘ought’ that I take to be the real ethico-politicial purchase and condition for a thoroughgoing sense of the transcendental (see the next two parts of this paper).

This allows me to respond to this corollary point: where does this place my approach in relation to approaches like ‘Foucault-inspired constructivist accounts’ and Stiegler’s approach to Kant in *Technics and Time 3*? (Zwier, this issue). The short response is that I take these approaches to evince a much stronger sense of the transcendental than postphenomenology, and to be highly relevant to expanding our sense of philosophy of technology; it’s just that I don’t think they ought to be assigned any kind of a priori explanatory exclusivity, because there are indefinitely many other approaches (past, present, and future) that have interesting contributions to make.^7^

Nothing logically necessitates a rehabilitation of the transcendental. Instead, it is a matter of engaging with the philosophies, artefacts and histories that are actually and contingently given, and of critically inquiring into the conditions of their ‘givenness’. This process can be as theoretical as it is practical, and we shouldn’t prejudge what counts in either respect. For it to occur at all, *actual* provocations to thought are required: enter, in my work, the concept of ‘exceptional technologies’ and an interest in Kant, post-Kantian philosophy, and the theme of the transcendental.

## Entailment

Zwier’s second big question is: ‘what does Smith’s renewal of the transcendental imply and entail?’ Let me expand on the answer just given: nothing logically necessitates rehabilitating the transcendental, and this, I think, *is what makes it valuable.* Stated differently: the entailment and implication at stake ought to be ethico-political and historical in the first instance. That is: it ought to be reflexively grounded in the contingency of events and emergences, rather than the logic of an underlying philosophical system worked out in advance.^8^

The verb I used in my initial paper was not ‘rehabilitate’ or ‘renew’, but ‘trivialise’. I stated that we should be ‘trivialising the transcendental’, and meant it in the sense of ‘demystifying’ or ‘making accessible’. Opting for this term over the others, I think, is no mere distinction without a difference.

‘Rehabilitate’ and ‘renew’ invite this question: ‘*for whom?’* One response is: *‘for those who have a sense of what ‘the transcendental’ is about, and that ‘it’ is a contested term in philosophy’*. On this interpretation, ‘rehabilitation’ and ‘renewal’ ultimately seem to have professional philosophers or a particular sub-group thereof as their ‘for whom’ (for instance: philosophers of technology).

If what I was arguing for amounted to this, it might deserve calling out as something potentially even cruder than the caricatural Hegelianism outlined at the end of the previous part. This is because it would emerge as little more than a changing of philosophical fashions. It would be as if all I was saying was this: *‘there was an empirical turn, now let’s have a transcendental one’.*

But this is not what I am saying, because it sticks to a logic of turns on the road (see Smith 2018: 1–6). The issue I want to get at, in contrast, is this: how can we get *off road* from philosophical fashions and their various dialectical ‘turns’, to a point where thoroughgoing philosophical engagement with the complexity of matters concerning technologies is just a matter of educational course?

This gets at the *‘for whom?’* (or better, *‘with whom?’*) of trivialising. Framed as a question: for whom do I think the transcendental motive to ‘go meta’ should be trivialised? Quite simply: *for everyone*, as a desirable habit of resilient, reflexive and well-educated existence in technologically-mediated societies.

It might be objected that such an objective is quixotic. I very much beg to differ. On the contrary, I think there are complex events and emergences to do with technologies at this historical juncture that are ethically and politically compelling us to try to put more of something like this into our theory and practice, at all sorts of educational levels, across all sorts of different educational platforms and contexts.^9^

This is the kind of *educative* implication and entailment I would like to see follow from a thorough trivialising of the transcendental (and a trivialising of all sorts of other philosophical concepts, thinkers, traditions and methods as well). Faced with our received institutions (and inertias) of education, this assuredly has the look of something improbable. It does not follow that it is impossible, undesirable or without value.

## Shared Agonism

Barker writes:

Perhaps it is agonism, struggle, the competition against conditions, that is at stake in Smith’s thinking? (After all there is a lot about games in there already.) In particular, Smith gives us Floridi’s example of the philosopher as a designer, as a figure who creatively designs solutions to a problem. I would like to slightly reword this as someone who creatively struggles with, agonizes over, a problem, rather than ‘solves’ or provides ‘solutions’ (Barker, this issue).

In the final part of my paper, I presented something like an allegory of agonism through an elaboration on Morton’s carpark example. With far greater clarity, Barker has named what I was agonising over (not without irony!): the status of agonism, not merely in philosophy of technology, but philosophy per se.

What grabs me about Barker’s point is the emphasis on agonising over problems. Before reading his response, my instinct when faced with ‘agonism’ would have been to think: ‘political philosophy’, ‘dispute’, ‘conflict’. What has never really struck me about the term, I am not ashamed to admit, is its proximity to ‘agonising’ and ‘agony’. Barker’s point made these senses hit home. More than that, however, he made me reflect on how such ‘agonising’ and ‘agony’ can be *shared*.

I have been critical of approaches including postphenomenology in this paper, and have responded to Zwier and Barker frankly. This all corresponds to the received philosophical sense of ‘agonism’ as connoting struggle. What underlies this sense as a condition of possibility—although this can all too often be forgotten when dispute and competition become vain ends in themselves—is the sense of ‘agony’ and ‘agonising’ occurring in relation to *shared* problems.

Imagine philosophy itself as a kind of automated vehicle. There is a tendency throughout contemporary cultures to (barely) ‘tolerate’ philosophy in this way: as a vehicle smoothly playing out various well-rehearsed turns on its own obscure little side track*. It is agonising to be ‘tolerated’ in this way.* Taking exception to this, and building on Barker’s point concerning agonism, I would like to propose something different: thoroughgoing philosophical education as a kind of historically-informed agonism capable of responding to emergent ‘off road’ technological problems.^10^ There are two key presuppositions here: first, that we are not lacking in such problems; second, that *all* the philosophical approaches mentioned in this paper, plus indefinitely many more, have something to offer in sharing this agony (including postphenomenology!).

## Footnotes

1 See footnote 7 below.

2 What I mean by ‘positivism’ and ‘presentism’ is related here. Positivism presupposes the natural sciences to offer our best possible explanatory framework. What I mean by ‘presentism’ (although this is admittedly a more nebulous term) is the tendency of certain philosophical approaches (in contemporary analytic philosophy, for instance) to discount thoroughgoing engagement with the history of philosophy. Both positivism and presentism can be perfectly consistent in and of themselves. What is ‘wrong’ with them is that, like postphenomenology (which I think evinces both tendencies (see Smith 2018: 27–33)), they block a metaphilosophically developed sense of the transcendental as a way of responding to new historical events and emergences (see below).

3 A more vexed issue is whether postphenomenology’s sense of the transcendental runs into a *de jure* limit by virtue of its focus on ‘mediation’ and *experience as such*, insofar as this excludes whole classes of technologies, systems and processes that act as constitutive conditions for technological objects and systems. It is my sense that many of the main approaches in philosophy of technology (postphenomenology, but also critical constructivism, and Actor Network Theory) are unequipped, for instance, to deal with the complex ontological, political and economic issues concerning what constitutes ‘uses’ and ‘usership’ on the Internet today by virtue of their tendency to focus on interfaces rather than databases. Authors like Fuchs (2016), Dean (2009), Floridi (2019), and Chun (2016) are, I think, much better equipped for engaging these issues, and have urgent contributions to make to our sense of what constitutes ‘philosophy of technology’ as a field.

4 See Hofstadter (1999), Williamson (2007), and Floridi (2011: 58-60).

5 Consider, for instance, how Kant’s reflections on the transcendental emerge from a consideration of Hume and Leibniz alike (2000). It should also be emphasised here that I take these points to hold for philosophers who are putatively ‘anti-systemic’ (for instance: Kierkegaard).

6 Stated differently: I locate the transcendental with actual processes, not ‘real’ ones in Hegel’s sense (of ‘rational’, ‘absolute’ and ‘teleological’). What I am after is much closer to Adorno’s ‘negative dialectics’ (1990), Deleuze’s sense of ‘transcendental empiricism’ (2004), or Nietzsche’s sense of ‘critical history’ (1999).

7 For instance: Floridi’s engagement with Kant (2019), but also Foucault’s own engagement, not merely with Kant (1991), but also with ancient approaches to ethics in his work on ‘technologies of the self’ (1998). Additionally, I have in mind work by thinkers such as Hui on Chinese philosophy of technology (2016), as well as work by Åsberg and Braidotti (2018) on feminist perspectives, Van Den Eede’s work on Bateson (2019), and Coeckelbergh’s on Wittgenstein (2017). Just as my work has involved an engagement with Kant, these approaches make a virtue of expanding the purview of philosophy of technology through appeal to different histories. Consider, in contrast, how three main currents in philosophy of technology today engage with the history of philosophy: Ihde’s postphenomenology, Feenberg’s critical constructivism, and Latour’s Actor Network Theory. Early Heidegger and parts of Husserl are the key historical references for Ihde; Marcuse is a key reference for Feenberg, and, while ANT’s references range all over the map, Kant is Latour’s key philosophical nemesis (1993). Polemically, the point is this: there can be much more to the constellation of thinkers informing philosophy of technology today than ‘early Heidegger + Husserl + Marcuse + [not] Kant (this last ‘not’ intended as an injunction, not a contradiction)’. Although this point is exaggerated, there is, I think, more than a grain of truth to it.

8 Zwier asks: ‘would a transcendental analysis of *our* ways of taking exception be relevant, and if so, how would Smith propose such an analysis is to be carried out?’ (Zwier, this issue). I take such reflexive analysis to be *highly* relevant. The remarks in this part are meant to say something about how it might be carried out.

9 Automated vehicle development and the Anthropocene, as developed in my initial paper, are two such issues. To cite a few others: critical engagement with forms of technological solutionism demonstrated during the 2020 COVID-19 pandemic; issues concerning technology and intergenerational justice; reflection on the differences between data, information, knowledge and wisdom (Kitchin 2014); critical reflection on forms of reasoning by analogy and disanalogy between historical and contemporary examples (Smith 2018: 77–105).

10 This is not to say that we should be rejecting tried and tested philosophical desiderata (proofs for the existence of God, or thought experiments, for instance). It is to say that we should be putting the skills they foster into contact with emergent problems. See, for instance, the treatment of the Trolley Problem in part two of my paper, and footnote 9 above.
